# Evidence for a double mutualistic interaction between a lizard and a Mediterranean gymnosperm, *Ephedra fragilis*

**DOI:** 10.1093/aobpla/plz001

**Published:** 2019-01-11

**Authors:** Francisco Fuster, Anna Traveset

**Affiliations:** Global Change Research Group, Institut Mediterrani d’Estudis Avançats (CSIC-UIB), Mallorca, Balearic Islands, Spain

**Keywords:** Balearic Islands, double mutualism, lizard pollination, opportunistic nectar-feeding, saurochory

## Abstract

An increasing number of double mutualisms (i.e. two interacting species benefiting each other in two different functions, e.g. pollination and seed dispersal) have been reported, mainly from island ecosystems, although we still lack much information on how effective such species are in both processes. Here, we assessed the pollination effectiveness of a double mutualism between an ancient Mediterranean gymnosperm, *Ephedra fragilis*, and a lizard, *Podarcis lilfordi*. On the one hand, we assessed the lizard contribution to different fitness measures (seed set and germination success), relative to that of insects and the wind effect; on the other, we determined the lizards’ seed removal rate (i.e. the quantity component of seed dispersal effectiveness). In both processes, we further tested for differences in their contributions among male, female and juvenile lizards. *Ephedra fragilis* showed to be mostly anemophilous, lizards and insects playing only a minor role on seed set. However, lizards qualitatively contributed to pollination success, as seeds coming from lizard-pollinated cones germinated at higher rates than those pollinated by wind or insects, although this was detected only for small seeds (<8 mg). The plant produced a low seed set (c. 23 %), which was compensated by a high seed germinability (c. 70 %). Adult male lizards were those most implicated in pollination, quantitatively more important than insects, and in seed dispersal. This work, thus, reports the importance of a lizard species in one of the few double mutualisms found in the World involving a gymnosperm, and it represents the first documentation of a double mutualism in the Mediterranean region. Our findings further contribute to highlight the role of both inter- and intraspecific differences in the effectiveness of mutualistic interactions.

## Teaser

Our study provides evidence that the relationship between the Balearic endemic lizard *Podarcis lilfordi* and the plant *Ephedra fragilis* constitutes one of the five pollination/seed dispersal double mutualisms found around the World between an animal and a gymnosperm, besides representing the first double mutualism reported in the Mediterranean region. Although *P. lilfordi* does not contribute substantially to the pollination of *E. fragilis*, it does play a crucial role in the dispersal process. In turn, by means of the pollination drops and the fleshy cone scales, the plants feed its only seed disperser in this islet.

## Introduction

Most plants that depend on animal pollination and seed dispersal are often served by different taxa for each of these ecological functions (Herrera and Pellmyr 2002). Those cases in which the same plant species offers both floral and fruit/seed resources to the same animal species, potentially acting as pollinators and seed dispersers, have been much less documented (e.g. Soriano and Ruiz 2002; Kelly *et al.* 2004; Hansen and Müller 2009a; García *et al.* 2012). Such phenomenon is known as ‘double mutualism’ (Hansen and Müller 2009a) and it appears to be especially frequent on island ecosystems, although a number of reports are also from mainland areas (Fuster *et al.* 2018). One likely reason for the major prevalence of double mutualism on islands is the presence of species that compensate their densities, i.e. have high population abundances due to the relatively lower species richness, and thus lower interspecific competition compared to mainland systems (Mac Arthur *et al.* 1972). The density compensation results into high intraspecific competition which in turn leads to a trophic niche expansion of the species, i.e. explores and uses new kinds of food items (Olesen and Valido 2003; Traveset *et al.* 2015). Examples of trophic niche expansion have been observed in lizards, geckos, or even iguanas, which are most often carnivores or insectivores in the mainland, but consume floral and fruit resources in many islands, acting as potential pollinators and seed dispersers of a wide variety of plant species (Olesen and Valido 2003). Reptiles have indeed been found as important potential double mutualists worldwide, mainly in island ecosystems (Fuster *et al.* 2018).

Vertebrates are the taxa most involved in double mutualism, although much remains to be investigated regarding how effective they are both as pollinators and seed dispersers of the same plant species (Fuster *et al.* 2018). Studies in which both pollination and seed dispersal in the same species are simultaneously analysed are indeed rather scarce (Soriano and Ruiz 2002; Kelly *et al.* 2004; Nyhagen *et al.* 2005; Hansen and Müller 2009a; García *et al.* 2012; Gomes *et al.* 2014). Moreover, despite the increasing number of studies reporting vertebrates as opportunistic nectar consumers (Traveset and Sáez 1997; Banack 1998; Olesen and Valido 2003; Ortega-Olivencia *et al.* 2005; Sazima *et al.* 2005; Le Péchon *et al.* 2013; da Silva *et al.* 2014; Traveset *et al.* 2015; Zoeller *et al.* 2016), still few evaluate the quantitative and qualitative component of pollination effectiveness (but see Rodríguez-Rodríguez and Valido 2008; Hansen and Müller 2009a; Ortega-Olivencia *et al.* 2012; Rodríguez-Rodríguez *et al.* 2013; Hervías-Parejo and Traveset 2018; Ratto *et al.* 2018). Such information is relevant, especially if we want to foresee the consequences of potential mutualistic disruptions due to the different drivers of global change (Toby Kiers *et al.* 2010).

Islands, in particular, are ecosystems highly sensitive to invasive species and species extinction (Sax and Gaines 2008), and the disruption of double mutualisms in these ecosystems might have negative consequences both at the species and community level (Traveset and Richardson 2014). If an animal double mutualist is locally extinct, or even if its abundance declines dramatically, both its pollination and seed dispersal functions will be lost simultaneously. Depending on the interaction strength between the partners, a mutualistic disruption can notably jeopardize plant success (Hansen and Müller 2009b; Bissessur *et al.* 2017). A recent study has shown that double mutualisms can belong to the core of the pollination–seed dispersal network (Olesen *et al.* 2018), which implies that they play an important role in community structure and function. Their disruption, thus, might increase the fragility of the network and cascade into further extinctions, especially in communities with depauperate faunas (Kaiser-Bunbury *et al.* 2010).

In this study, we analyse the potential double mutualistic interaction between a species of lizard, *Podarcis lilfordi*, and the ancient shrub *Ephedra fragilis*. This plant species belongs to the gymnosperms, thus producing neither flowers nor fruits. Instead, ovules are located within cones which, in some cases, develop into fleshy fruitlike structures. Most gymnosperm species produce a solution on the cone that allows capture pollen from the air, and are thus wind-pollinated (Niklas 1982, 1985; Labandeira *et al.* 2007; Nepi *et al.* 2017; Walas *et al.* 2018). Although most *Ephedra* species are wind-pollinated (Niklas and Buchmann 1987 and references therein; Pellmyr 2002; Bolinder *et al.* 2016), some of them (*E. fragilis*, *E. foeminea* and *E. aphylla*) are also pollinated by animals (Bolinder *et al.* 2015a, b; Celedón-Neghme *et al.* 2016). Thus, besides capturing pollen from the air and aiding in pollen germination, these pollination drops can be attractive to animals, since they are rich in sucrose and contain amino acids and proteins (von Aderkas *et al.* 2015; Nepi *et al.* 2017). These secretions are produced by the nucellar cells in pistillate cones, i.e. female plants (Takaso and Owens 1995). However, in the case of *E. fragilis*, as also observed in *E. foeminea*, both functional ovules in pistillate cones and non-functional ovules in staminate cones produce pollination drops, thus attracting animals to both male and female plants (Celedón-Neghme *et al.* 2016). Although, both insects and lizards have been reported to visit the cones of *E. fragilis*, their contribution to pollination success is unknown. Moreover, lizards also ingest and efficiently disperse the seeds of *E. fragilis* when feeding on its fleshy cone scales (Rodríguez-Pérez *et al.* 2012; Neghme *et al.* 2017). Hence, the lizard–plant mutualistic relationship constitutes a potential double mutualism. Our main aim here was to assess quantitative and qualitative components of the effectiveness (*sensu*Schupp *et al.* 2017) of such double mutualistic interaction. For that, we evaluate the importance of lizards for plant pollination success, in terms of visitation rate, seed production and seed germinability, and compared it to that of insects and wind pollination. Moreover, based on our preliminary observations, we wanted to determine whether there are differences, both in pollinating plant visitation and seed consumption, between lizards of different sexes and ages, as these are known to have different behaviour which might influence both the pollination and seed dispersal processes (Rodríguez-Rodríguez *et al.* 2013; Pérez-Méndez *et al.* 2018).

## Methods

### Study site and species

This work was conducted at Sa Dragonera Natural Park (39°35′N, 2°19′E), a 288-ha islet located at c. 800 m of the western coast of Mallorca Island, Balearic Islands, in the Western Mediterranean Sea. The islet has an annual precipitation of 350 mm and average annual temperatures ranging from 17 to 18 °C (Spanish Agency of Meteorology, www.aemet.es). The study site was located at the north-eastern tip of the islet, a rocky coastal area with a vegetation dominated by shrubs of *E. fragilis* and *Pistacia lentiscus*, mixed with *Olea europaea*, *Phillyrea angustifolia* and *Cneorum tricoccon*.

*Ephedra fragilis* (Ephedraceae) is a dioecious evergreen shrub, up to 4 m in height, distributed in the Western Mediterranean basin (Markgraf 1964). Besides the Balearic Islands, it is present in the southern Iberian Peninsula, in some points of northern Africa, and also in some of the Canary Islands (specifically, in La Palma and Tenerife). It prefers calcareous or gypsum arid places, salty sandy areas and sclerofilous scrublands, from 0 to 1100 m in elevation (do Amaral-Franco 1986). The plant does not produce cones every year, and it shows years of mast cone production. Both male and female individuals develop cones that secrete a pollination drop, a sugar-rich solution produced to capture pollen from the air, but which is also consumed by both lizards and insects (Celedón-Neghme *et al.* 2016). Female cones become fleshy after the pollination period, producing red or yellow fruitlike structures, each bearing only one seed. *Ephedra fragilis* has been reported to be dispersed by birds in the mainland (Herrera 1987), as well as on the island of Mallorca (mostly by *Sylvia melanocephala*; A. Traveset and J. P. González-Varo, pers. obs.), where lizards are no longer present since they became extinct after the introduction of predators (Pérez-Mellado 2002). In Dragonera Island, however, only lizards have been observed so far feeding on the female cones of this plant (pers. obs.).

*Podarcis lilfordi* (Lacertidae) is an endemic lizard to the Gymnesic Islands (eastern Balearic Islands), i.e. Mallorca, Menorca and surrounding islets. It currently survives only in the islets, given that it disappeared from the largest Mallorca and Menorca after the introduction of carnivorous mammals (Pérez-Mellado 2002). It frequently feeds on floral resources and fruits (Pérez-Mellado and Traveset 1999), acting as a legitimate pollinator (e.g. Traveset and Sáez 1997; Celedón-Neghme *et al.* 2016) and seed disperser of different species (e.g. Rodríguez-Pérez and Traveset 2010; Rodríguez-Pérez *et al.* 2012; Celedón-Neghme *et al.* 2013; Neghme *et al.* 2017). Adult lizards are distinguishable from juveniles by their larger body size, as well as adult males are also distinguishable from adult females by their larger and more robust body and head (Salvador 2009).

### Plant observations of potential pollinators and seed consumers

Both in 2015 and 2016, we observed visitors to the cones of *E. fragilis* during its pollination period, from early May to early June. All observations were made from 8:00 am to 18:30 pm on a total of 23 different female plants (13 and 10 individuals in 2015 and 2016, respectively) and 22 male plants (11 individuals every year), accumulating a total of ≈35 and 51 h in 2015 and 2016, respectively. In every census (15–20 min long), we counted the number of lizards—distinguishing between juveniles, females and males—and insects visiting the plant and touching the cones, the time that each individual spent on them, as well as the number of cones available on the plant. Insects were captured for further identification with a reference pollinator collection available at the Mediterranean Institute of Advanced Studies (IMEDEA CSIC-UIB). We further wanted to estimate the quantity of pollen transported by lizards. For this purpose, we obtained 18 pollen samples—11 from male lizards and 7 from females—from lizards we captured using a noose. A small cube of glycerine jelly tinged with fuchsine was swabbed on their snout and around the head; the jelly cube was then placed on a slide, melt with heat, and finally sealed with transparent nail polish. The slides were taken to the lab and analysed with a Leica light microscope at 10× and 40× magnification.

During July 2016, we also observed seed consumers on 12 female individuals, within the same daytime period and for a total of ≈28 h. In each census, we recorded the number of fleshy female cones removed by juveniles, females and males visiting each plant, counting the number of fleshy female cones in each plant before the census.

### Pollinator exclusion experiment

**Figure 1.**
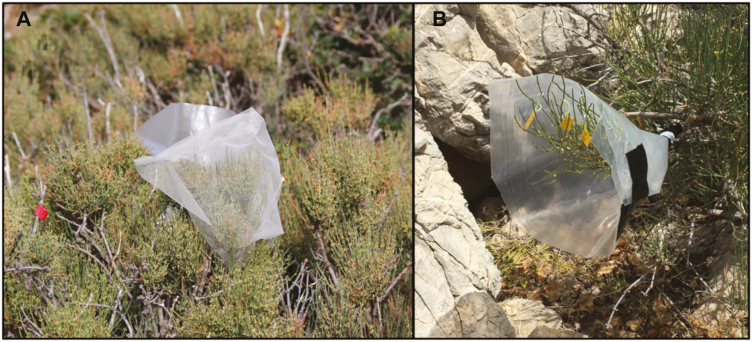
Anemogamy A) and lizard exclusion B) treatments carried out in *Ephedra fragilis* on Dragonera Island during the pollination season of 2016.

Between the end of April and end of July 2016, we conducted an exclusion experiment to quantify the contribution of lizards, insects and wind on plant pollination success. On each of 15 female individuals, we set up three treatments: (i) *insect pollination*: branches were surrounded with a plastic cone that impeded lizard access but allowed insects to visit the cones (and thus allowed also wind pollination although only through the upper part; see Fig. 1); (ii) *wind pollination*: branches were bagged with a bridal veil bag (mesh size: 1 mm) that allowed pollen to pass through but excluded both insects and lizards; and (iii) *control*: branches were simply tagged but left open to pollination, being the only treatment with lizard contribution. Once cones were no longer receptive (when they begin to ripen), we bagged control and insect pollination branches with cloth bags to avoid seed removal or drop before seed set could be recorded. At the end of July, we collected the mature female cones from every treatment to obtain seed set. Seed set was thus considered as the total number of seeds produced, discarding aborted cones that did not develop into seeds, relative to the number of initial female cones. Aborted cones remain very tiny and greenish/yellowish and are thus quite distinguishable. We removed the seeds in the laboratory and weighted them (20 per branch) with an electronic balance (0.1 mg precision). For the seed germination experiments, we individually weighted and sowed 200 randomly selected seeds per treatment, planting thus a total of 600 seeds. All seeds were sown in late September in germination trays, filled with universal substrate and watered every 2–3 days. Apex emergence (germination hereafter) was recorded every 2–3 days until the germination stopped.

### Data analysis

All data used in this work are included as (abcdefghlink)Supporting Information(abcdefghxref). Data on lizard and insect visitation to plants (for either pollination or seed dispersal) were analysed by means of generalized linear mixed models (GLMMs), using a gamma error distribution with a logarithmic link function. In the case of pollination, pollinator group (insects, male, female and juvenile lizards), year (2015 and 2016), plant sex (male or female) and the interaction between these three variables were included as fixed effects. In order to control for floral display, which might influence pollinator attraction, we included the total number of cones per plant as offset; census ID was nested within individual plant and included as random effect in the model. In the case of dispersal (i.e. fleshy female cone consumption rates), we used lizard group (male, female and juvenile lizards) as fixed effect, number of fleshy female cones per plant as offset, and again census ID nested within individual plant as random effect. Year was not included in this case, as censuses were done only in 2016. The number of pollen grains transported on male and females were additionally compared by means of an ANOVA after a data normalization using the square root of pollen grains counted.

Seed set and seed weight were analysed fitting GLMMs with binomial and Gaussian error distributions, respectively. In both cases, we included treatment and plant size (height and width) in the models as fixed effects. Seed germination was also analysed fitting GLMMs with binomial error distribution, including treatment, seed weight and their interaction, and plant height and width, as fixed effects. In the three sets of models, plant ID was included as random effect.

All models used were ran with the ‘glmer’ function from ‘lme4’ package (Bates *et al.* 2015) in R (version 3.3.3.; R Development Core Team). We then used the Akaike information criterion corrected for small sample sizes (AICc; Zuur *et al.* 2009) to select the best models (those with lowest AICc values). This model selection was made using the ‘dredge’ function in the ‘MuMIn’ package (Barton 2016). Models with ΔAICc ≤ 2 were considered to be equivalent.

## Results

**Figure 2.**
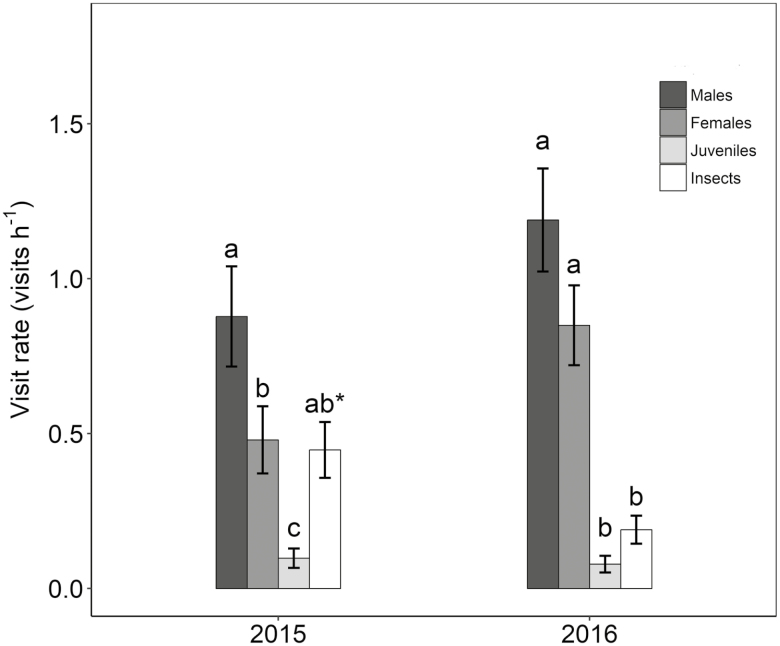
Mean and standard error (SE) of the plant visitation rate (visits per hour) of lizards and insects during the pollination periods of 2015 and 2016. Different letters above the columns indicate significant differences (Tukey’s *post hoc* tests, *P* < 0.05) among pollinator groups; differences are given for each year separately; *P < 0.001.

All models selected based on AICc and considered equivalents (ΔAICc ≤ 2) are summarized in Table 1. We found significant differences among pollinator groups in visitation rates, and such differences varied between years (Table 1). Overall, adult lizards, both male and female, visited cones more frequently than insects, especially in 2016 (Fig. 2). They also spent much more time on the plants (males: 10.59 ± 0.52 min h^−1^; females: 8.6 ± 0.5 min h^−1^; juveniles: 9.3 ± 1.4 min h^−1^) than insects (3.1 ± 0.4 min h^−1^), possibly visiting more cones. Lizards transported pollen both on their heads and bodies. We found that each individual carried an important quantity of pollen grains, which did not differ between sexes (males: 438 ± 140, *N* = 11; females: 421 ± 182, *N* = 7; ANOVA: *F* = 0.008, *df* = 1, *P* = 0.93). The insect species found on the *E. fragilis* cones included mostly flies (c. 10 sp.) but also some bees (2 sp.), ants (1 sp.), beetles (1 sp.), true bugs (1 sp.) and moths (1 sp.). All these insects are vouchered at the IMEDEA pollinator collection. Insects and lizards behaviour was different during the visits. Insects usually go straight to target cones after flying around the plant, touching only a small number of cones in each single visit. By contrast, lizards climb the plant and walk on the branches contacting many cones with their heads and body when trying to reach the nectar.

**Table 1.** Generalized linear mixed models selected based on AICc for the different response variables (models with ΔAICc ≤ 2 were considered to be equivalent). * Means significant effect.

Response variable	Model	Predictor variable	Random	Error distribution	Link function	*χ* ^2^	*df*	*P*
Plant visitation rate	1	Pollinator	Plant ID/census ID	Gamma	Log	201.09	3	<0.001*
		Year				8.03	1	0.005*
		Pollinator × year				34.40	3	<0.001*
	2	Pollinator	Plant ID/census ID	Gamma	Log	201.05	3	<0.001*
		Year				8.10	1	0.004*
		Plant sex				0.13	1	0.722
		Pollinator × year				34.37	3	<0.001*
Seed set	1	Treatment	Plant ID	Binomial	Logit	1670.9	2	<0.001*
	2	Treatment	Plant ID	Binomial	Logit	1670.88	2	<0.001*
		Plant width				1.28	1	0.258
	3	Treatment	Plant ID	Binomial	Logit	1670.89	2	<0.001*
		Plant height				0.63	1	0.426
Seed weight	1	Treatment	Plant ID	Gaussian	Identity	5.10	2	0.080
Germination %	1	Treatment	Plant ID	Binomial	Logit	3.25	2	0.197
		Seed weight				28.95	1	<0.001*
		Treatment × seed weight				12.65	2	0.002*
	2	Treatment	Plant ID	Binomial	Logit	3.01	2	0.222
		Seed weight				31.47	1	<0.001*
		Plant width				2.55	1	0.111
		Treatment × seed weight				12.01	2	0.003*
	3	Treatment	Plant ID	Binomial	Logit	3.20	2	0.202
		Seed weight				29.30	1	<0.001*
		Plant height				0.39	1	0.533
		Treatment × seed weight				12.60	2	0.002*

**Figure 3.**
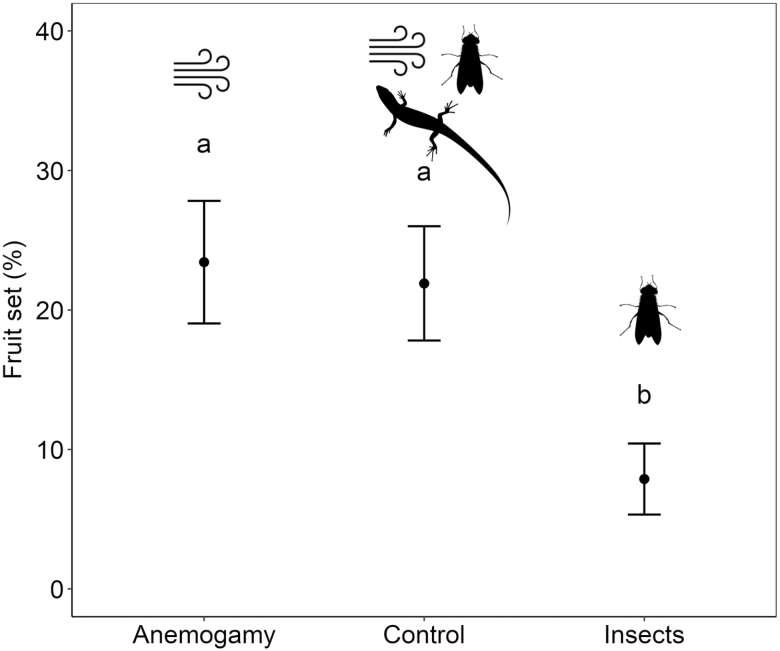
Mean and standard error (SE) of seed set (% seeds) of the different treatments. Different letters indicate significant differences (Tukey’s *post hoc* tests, *P* < 0.05) among treatments.

**Figure 4.**
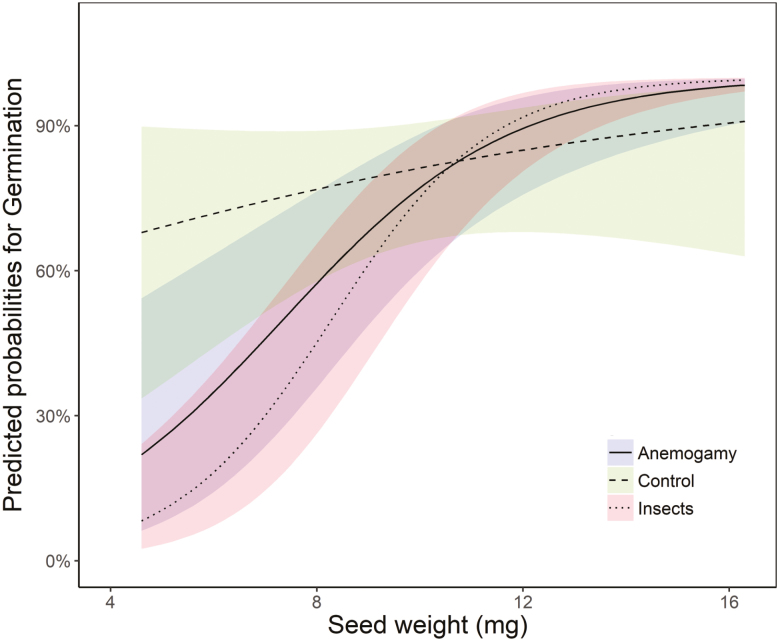
Interaction between seed germination and seed weight (predicted probabilities from the GLMM) in the three pollination treatments. Values show least squares means and confidence intervals.

Seed set resulting from the anemogamy treatment did not differ significantly from the control one (Fig. 3). In fact, seed set of the insect treatment (which also allowed some wind pollination, but much less than the anemogamy treatment) was only c. 10 % (Fig. 3). Hence, despite the high frequency of cone visits by lizards and the high diversity of insects, the fraction of cones that set seed is not raised significantly with animal pollination. Seed weight was similar among treatments (anemogamy: 9.85 ± 0.13 mg; insect pollination: 9.71 ± 0.16; and control: 9.62 ± 0.13 mg; *χ*^2^ = 5.05, *df* = 2, *P* = 0.08). Nevertheless, there was a significant interaction effect between seed weight and treatment on germination (*χ*^2^ = 12.65, *df* = 2, *P* = 0.002; Table 1). The germination probability predicted by the model (Fig. 4) showed a general increase when seeds were heavier; conversely, germination probability of the lighter seeds (<8 mg) was significantly higher in the control than in the anemogamy or insect pollination treatments, suggesting that lizards might contribute to increase germination of the light seeds.

**Figure 5.**
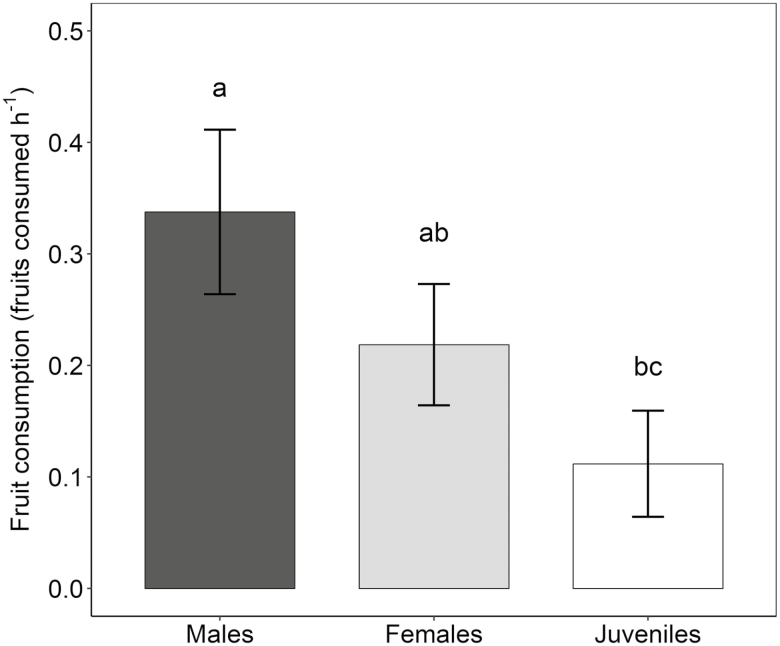
Seed consumption (fleshy female cones consumed per lizard and per hour) by lizards during the dispersal period of 2016. Different letters indicate significant differences (Tukey’s *post hoc* tests, *P* < 0.05) among lizard groups.

Regarding consumption rates of fleshy female cones, we detected significant differences among lizard males, females and juveniles (*χ*^2^ = 18.30, *df* = 2, *P* < 0.001). Males were the most frequent consumers (0.33 ± 0.07 fleshy cones consumed per hour), followed by females (0.22 ± 0.05) and juveniles (0.11 ± 0.05) (Fig. 5).

## Discussion

Our study provides evidence that this plant–lizard relationship constitutes one of the five pollination/seed dispersal double mutualisms found around the World between an animal and a gymnosperm (Fuster *et al.* 2018), besides representing the first double mutualism reported in the Mediterranean region. Although *E. fragilis* shows to be mainly pollinated by wind, we found that lizards, while obtaining energy resources from this gymnosperm, they are relevant for its reproduction as they (i) transport large amounts of pollen from male to female cones when feeding on pollination drops, (ii) increase the germination of light seeds and (iii) act as their main seed dispersers, at least on Dragonera Island (Rodríguez-Pérez *et al.* 2012; Neghme *et al.* 2017; this study). Our results do show a minor contribution of lizards to pollination success. This does not imply, however, that they are irrelevant in the pollination process, as germination of small seeds coming from cones visited by lizards did increase. Hence, although the mutualistic benefit provided by lizards is not the primary determinant of pollination success, our results nevertheless provide clear evidence that a quantifiable double mutualism does exist. Moreover, lizards are fed by the plant with pollination droplets, maintaining its only seed disperser. Thus, perhaps the distinction between single and double mutualism should not be considered strictly binary: a double mutualism can be essential for the survival of one partner but merely beneficial to the other.

Anemogamy is the main pollination system within gymnosperms, although entomophily has been documented in some species (Bolinder *et al.* 2015a, b; Labandeira *et al.* 2007; Hall and Walter 2018). Mixed pollination systems, i.e. wind and insect pollination, are also present in some gymnosperms (e.g. Kono and Tobe 2007; Gong *et al.* 2016; Hall and Walter 2018; Walas *et al.* 2018). Within the *Ephedra* genus, only *E. foeminea* has been reported as insect-pollinated (Bolinder *et al.* 2015a, b), while *E. aphylla* is known to have a mixed pollination system (Meeuse *et al.* 1990). Our study with *E. fragilis* confirms the previous finding by Celedón-Neghme *et al.* (2016) that this species also has a mixed pollination system, going even further and showing that lizards play a more important role than insects in the pollination process. Evolving a mixed pollination system might be a response to the unpredictability of a single pollination vector, e.g. low pollinator density, or low wind (Ríos *et al.* 2014 and references therein; da Costa *et al.* 2018).

Our results showed that lizards play a more important role than insects in the quantity component of pollination effectiveness. These findings are consistent with those from other systems in which lizards and insects act as pollinators (e.g. Hansen *et al.* 2007; Gomes *et al.* 2014). One plausible explanation is that lizards contact many more cones (and flowers in other species) with their bodies while foraging for pollination drops (nectar in other species) than insects which usually go straight to the target cone. Lizards, in fact, stayed on *E. fragilis* plant about three times more than insects, similar to what has been found in other species like *Euphorbia dendroides* (Traveset and Sáez 1997). Thus, lizards’ behaviour and their longer time on the plant may well lead to higher pollen removal and higher pollen deposition in male and female plants, respectively.

The low seed set (c. 25 %) of *E. fragilis* is at least partly compensated by the high seed germination (c. 70 %) which might contribute to maintain the population. Although mature seeds might not necessarily be fertile (i.e. with a full embryo inside), the different treatments, which only modify the pollinator agent, were performed in the same plant individuals; thus, the differences observed are attributed to the pollinator agent. The significantly lower seed set found in the insect pollination treatment (which includes also wind pollination) compared to the anemogamy treatment (only wind pollination) suggests that insects may be having an effect that is more negative than beneficial for pollination success. It is likely that by feeding upon the pollination drops they actually reduce the probability of pollen germination or simply that they are not depositing as much pollen grains as wind does. Although we did not observe any insect exit hole on the seeds, oviposition of eggs by parasitoids have been found in other Mediterranean *Ephedra* species (Askew and Blasco-Zumeta 1997). Alternatively, the plastic cone used to exclude lizards in the insect pollination treatment might have partly affected the seed set results (by reducing the additional effect of wind-dispersed pollen in such treatment). In contrast to our results, Celedón-Neghme *et al.* (2016) reported cone visits by animals (pooling lizards and insects) to *E. fragilis* to slightly increase seed set, what suggests that the importance of animals for seed production may vary across years, probably depending on the effects of factors like wind intensity, insect abundance and food availability for lizards.

Interestingly, lizards showed to influence seed germination but only for small seeds, i.e. when seeds were light (<8 mg). By contrast, large seeds resulting from all treatments germinated similarly. Small seeds would actually be expected in the habitats where *E. fragilis* is usually found, poor-resource sites, generally with low water availability. The better germinability of light seeds resulting from lizard pollination might indeed be relevant to assure the viability of the plant population. This higher germinability might respond to a better genetic load (González-Varo and Traveset 2010), which might be related to the distance to the pollen origin, although further research on this matter would be needed to test such hypothesis.

Interestingly, not all lizard individuals showed to play a similar role in the double mutualistic relationship. Adults were more frequent cone visitors and seed consumers than juveniles. These differences might respond to different energetic requirements, since older individuals of omnivorous lizards use to feed on more plant material than juveniles (e.g. Durtsche 2000; Fialho *et al.* 2000). Juveniles may need higher energy resources to grow and avoid to be predated, and thus insects constitute a more profitable food. By contrast, adults, with lower energy requirements, can include nectar or fruit in their diet, easier accessible food, which allows saving energy required to hunt insects. Although gymnosperms do not provide nectar, the pollination drops contain sugars (generally in low concentrations), amino acids and proteins which can be profitable for lizards (Nepi *et al.* 2009, 2017). In the case of *E. fragilis*, however, pollination drops present high sugar concentrations (Celedón-Neghme *et al.* 2016), similarly to *E. distachya* (Ziegler 1959). Whether such higher sugar concentrations are due to abiotic factors or are the result of a process of selection by either insects, lizards or both is unknown. Intraspecific differences in either the quantitative or qualitative component of pollination have been reported in other plant–lizard systems (e.g. Nyhagen *et al.* 2001; Rodríguez-Rodríguez *et al.* 2013; Gomes *et al.* 2014). During the seed dispersal phase, differences were also found in fleshy female cone removal rates, similarly observed in another shrub, *C. tricoccon*, which coexists with *E. fragilis* on Dragonera Island. In this last system, male adult lizards often displace females and juveniles from fruiting plants (F. Fuster, pers. obs.). Adult lizards, mostly males, are thus the most important seed consumers, and given that they can disperse larger seeds than juveniles, they probably contribute to a higher seed germinability in the *E. fragilis* population. Such intraspecific differences in seed removal have been reported in various systems (e.g. the endemic Canarian *Neochamaelea pulverulenta*; Pérez-Méndez *et al.* 2018) and should be considered when elucidating the ‘forbidden links’ in plant–frugivore interactions (see González-Varo and Traveset 2016). A low value of seed removal rate by juveniles might be simply due to their incapacity of ingesting large fleshy cones. Intraspecific resource segregation can actually be considered a way to avoid the high intraspecific competition usually found in island ecosystems, because of the high animal densities of the same species and few available resources. Such resource segregation has already been observed in different animal groups (e.g. Leung *et al.* 2012; Miranda *et al.* 2012; Wan *et al.* 2013; Mata *et al.* 2016), including lizards (Kaliontzopoulou *et al.* 2012), and might be more common than previously thought. It is thus important to assess the intraspecific differences of mutualist partners, as such information can be useful to better understand the functioning of the ecological process in question (González-Varo and Traveset 2016; Zwolak 2018).

Both partners of the double mutualistic interaction described here show an important dependence on each other: the plant seems to be a principal food source for the lizard, both during the pollination and the seed dispersal season; *E. fragilis* is actually one of the few species fruiting during July. In turn, lizards contribute to the pollination success of the plant, although more qualitative than quantitatively by favouring the germination capacity of the light seeds produced, and the plant depends mostly on lizards for seed dispersal (as previously mentioned, we never saw a bird or other animal groups removing fleshy cones in Dragonera). In this island, *P. lilfordi* has further been found to contribute with higher recruitment and seedling survival compared to non-dispersed seeds (Neghme *et al.* 2017).

In short, although *P. lilfordi* does not contribute substantially to the pollination of *E. fragilis*, it does play a crucial role in the dispersal process. In turn, by means of the pollination drops and the fleshy cone scales, the plants feed its only seed disperser in this islet. From the plant’s perspective, thus, the tight plant–lizard interaction represents a risk, as any decline in the lizard population density would affect the functioning of the double mutualism, probably with detrimental consequences for plant reproductive success and survival in the long term (Traveset and Riera 2005; Traveset *et al.* 2012). Breakages in double mutualisms are already being reported (e.g. Hansen and Müller 2009b; Bissessur *et al.* 2017). Given the fragility of insular ecosystems like Dragonera (Traveset *et al.* 2013; Traveset and Richardson 2014; Bellard *et al.* 2017), the understanding of the functioning of their communities, including the presence of complex interactions such as double mutualisms, becomes a much necessary task.

## Sources of Funding

This work is framed within projects CGL2013-44386-P and CGL2017-88122P, and F.F. was funded by a PhD fellowship (BES-2014-068207), all financed by the Spanish Government.

## Contributions by the Authors

F. F and A. T. designed research. F. F. conducted the fieldwork, analysed data, and wrote the manuscript with important A. T. contribution.

## Conflict of Interest

None declared.

## Supporting Information

The following additional information is available in the online version of this article—

File S1. Pollination censuses data.csv.

File S2. Exclusion data.csv.

File S3. Pollen samples.csv.

File S4. Sow data.csv.

File S5. Dispersion data.csv.

Supporting Information
